# Ginkgolide C Suppresses Adipogenesis in 3T3-L1 Adipocytes via the AMPK Signaling Pathway

**DOI:** 10.1155/2015/298635

**Published:** 2015-08-30

**Authors:** Chian-Jiun Liou, Xuan-Yu Lai, Ya-Ling Chen, Chia-Ling Wang, Ciao-Han Wei, Wen-Chung Huang

**Affiliations:** ^1^Department of Nursing, Chang Gung University of Science and Technology, 261 Wen-Hwa 1st Road, Kwei-Shan, Taoyuan, Taiwan; ^2^Department of Nutrition and Health Sciences, Chang Gung University of Science and Technology, 261 Wen-Hwa 1st Road, Kwei-Shan, Taoyuan, Taiwan; ^3^Graduate Institute of Health Industry Technology, Chang Gung University of Science and Technology, Kwei-Shan, Taoyuan, Taiwan; ^4^Research Center for Industry of Human Ecology, Chang Gung University of Science and Technology, Kwei-Shan, Taoyuan, Taiwan

## Abstract

Ginkgolide C, isolated from *Ginkgo biloba* leaves, is a flavone reported to have multiple biological functions, from decreased platelet aggregation to ameliorating Alzheimer disease. The study aim was to evaluate the antiadipogenic effect of ginkgolide C in 3T3-L1 adipocytes. Ginkgolide C was used to treat differentiated 3T3-L1 cells. Cell supernatant was collected to assay glycerol release, and cells were lysed to measure protein and gene expression related to adipogenesis and lipolysis by western blot and real-time PCR, respectively. Ginkgolide C significantly suppressed lipid accumulation in differentiated adipocytes. It also decreased adipogenesis-related transcription factor expression, including peroxisome proliferator-activated receptor and CCAAT/enhancer-binding protein. Furthermore, ginkgolide C enhanced adipose triglyceride lipase and hormone-sensitive lipase production for lipolysis and increased phosphorylation of AMP-activated protein kinase (AMPK), resulting in decreased activity of acetyl-CoA carboxylase for fatty acid synthesis. In coculture with an AMPK inhibitor (compound C), ginkgolide C also improved activation of sirtuin 1 and phosphorylation of AMPK in differentiated 3T3-L1 cells. The results suggest that ginkgolide C is an effective flavone for increasing lipolysis and inhibiting adipogenesis in adipocytes through the activated AMPK pathway.

## 1. Introduction

Obesity and being overweight are a major public health problem in developing and developed countries [1]. Obesity is linked to an increased prevalence of chronic diseases including cardiovascular disease, type 2 diabetes, and cancer [2]. Adipocyte proliferation and lipid accumulation are main factors causing overweight, and excess nutrient intake results in lipid conversion and accumulation in adipocyte and liver cells [3]. Triglycerides, a major lipid category, consist of three fatty acids and glycerol [4]. Many studies have indicated that fatty acid and triglyceride synthesis rely on complex and multiple pathways. Important transcription factors including peroxisome proliferator-activated receptor (PPAR), CCAAT/enhancer-binding protein (C/EBP), and sterol regulatory element-binding protein 1c (SREBP-1c) can increase expression of genes for enzymes associated with fatty acid synthesis, leading to excessive lipid accumulation in adipose tissue and hepatocytes [5]. One group has reported that lipolysis enzymes can promote lipid breakdown and increase triglyceride metabolism to decrease lipid accumulation in adipocytes and hepatocytes [6]. One outcome of this activity could be to inhibit triglyceride synthesis as well as promote the decomposition of triglycerides in adipocytes and possibly ameliorate the obesity effect.

Recent studies have found that AMP-activated protein kinase (AMPK) is an important regulator of energy and AMPK activation is closely related to the balance between lipid accumulation and carbohydrate metabolism [7]. Phosphorylation of AMPK stimulates substrate phosphorylation of acetyl-CoA carboxylase (ACC), which provides malonyl-CoA substrate for biosynthesis of fatty acids [8]. However, phosphorylation of ACC does not lead to catalysis of acetyl-CoA to malonyl-CoA. In clinical medicine, metformin is used to treat type II diabetes and can increase lipolysis and block the formation of fatty acids and triglycerides [9]. Metformin also increases the activity of AMPK, leading to the suppression of ACC activity [10]. Thus, metformin could enhance AMPK activity and improve the excessive accumulation of triglycerides in adipocytes for reducing the prevalence of metabolic syndrome in diabetes patients.

*Ginkgo biloba* L. has been used as a medicinal herb for a long time in oriental and western medicine [11]. In China and Taiwan,* Ginkgo* leaf extract is applied to treat cardiovascular, dementia, and cerebrovascular diseases, and the fruit of* Ginkgo *is used to treat asthma [2, 12]. In recent years, several compounds have been isolated and purified from* Ginkgo*, including terpenoids, diterpene lactones, and polyphenols [13]. The diterpene lactones that exert a pharmacological effect are ginkgolides A, B, C, M, P, and Q [14]. Many studies have demonstrated that ginkgolides can enhance cognitive function, decrease atherosclerosis, and block platelet-activating factor [13, 15].* Ginkgo biloba* extract also has been reported to improve obesity and insulin signaling in obese rats [16]. In this study, we investigated whether ginkgolide C can modulate adipogenesis, lipolysis, and the AMPK signaling pathway in differentiated adipocytes.

## 2. Materials and Methods

### 2.1. Chemical Reagent

Ginkgolide C (purity ≥ 96% by HPLC) was purchased from Sigma-Aldrich (St. Louis, MO, USA) and dissolved in DMSO at stock concentrations of 100 mM. In all experiments, the final concentration of DMSO in culture was ≤0.1%.

### 2.2. Cell Culture

The 3T3-L1 murine preadipocyte cell line was purchased from the Bioresource Collection and Research Center (BCRC, Taiwan). Cells were routinely cultured in Dulbecco's modified Eagle's medium (DMEM) supplemented with 10% fetal calf serum at 37°C in a 5% CO_2_ atmosphere until adipocyte differentiation.

### 2.3. Cell Viability Assay

3T3-L1 cells were treated with various concentrations of ginkgolide C in 96-well plates for 24 h. Cell viability was analyzed by the MTT assay as previously described [17]. The culture medium was removed, and the cells were incubated with 100 *μ*L MTT solution (5 mg/mL, Sigma) for 4 h at 37°C. After plates were washed, isopropanol was added to dissolve formazone crystals, followed by absorbance detection with a spectrophotometer (Multiskan FC, Thermo, Waltham, MA, USA) at 570 nm.

### 2.4. Adipocyte Differentiation

3T3-L1 cells (10^4^/mL) were seeded in 6-well plates and adipocyte differentiation was induced as previously described [18]. Briefly, 3T3-L1 cells were cultured in DMEM containing 10% fetal bovine serum and stimulated with 1 *μ*M dexamethasone (Sigma), 0.5 mM 1-isobutyl-3-methylxanthine (Sigma), and 10 *μ*g/mL insulin (Sigma) for 2 days. Two days later, DMEM supplemented with 10 *μ*g/mL insulin was used as differentiation medium for 2 days, with changes every 2 days. On day 8, the differentiated adipocytes were treated with ginkgolide C (3–100 *μ*M).

### 2.5. Oil Red O Staining

Differentiated adipocytes were treated with ginkgolide C on a 3 cm plate for 24 h, and cells were fixed with 10% formalin for 30 min. Next, cells were washed with phosphate-buffered saline and stained with oil red O for 1 h, followed by observation of the oil droplets under microscopy (Olympus, Tokyo, Japan). Plates were washed and treated with isopropanol and the lipid accumulation was quantified by measuring absorbance at an optical density of 490 nm with a microplate reader (Multiskan FC, Thermo).

### 2.6. Measurement of Glycerol Production

Glycerol production was assayed using the Glycerol Assay Kit (Sigma) according to the manufacturer's protocol. The supernatants were collected to be added with reagent to a 96-well plate, and absorbance at 570 nm was measured using a microplate reader (Multiskan FC, Thermo).

### 2.7. Preparation of Proteins and Western Blot Analysis

3T3-L1 cells were treated with ginkgolide C in 6-well plates, and cells were lysed with protein lysis buffer containing protein inhibitor cocktail (Sigma). Protein doses were calculated using a BCA protein assay kit (Pierce). Equal amounts of the denatured protein were run on 8–10% sodium dodecyl sulfate-polyacrylamide gels, followed by transfer to polyvinylidene fluoride membranes (Millipore, Billerica, MA, USA). The membranes were blocked with 5% nonfat dried milk in TBST buffer (150 mM NaCl, 10 mM Tris-HCl pH 8.0, 0.1% Tween 20) for 1 h and then incubated overnight at 4°C with the following respective primary antibodies: phosphorylated-AMPK*α*, AMPK, fatty acid synthase (FAS), fatty acid-binding protein (aP2), SREBP-1c, and lipoprotein lipase (LPL) (Santa Cruz, CA, USA); phosphorylated acetyl-CoA carboxylase-1 (pACC-1), ACC-1, PPAR-*α*, PPAR-*γ*, C/EBP*α*, C/EBP*β*, phosphorylated hormone-sensitive lipase (HSL, pHSL), HSL, and adipose triglyceride lipase (ATGL) (Epitomics, Burlingame, CA, USA); sirtuin 1 (Sirt1) (Millipore); and *β*-actin (Sigma). The membranes were washed with TBST buffer and then incubated with secondary antibodies for 1 h at room temperature. Finally, membranes were treated with luminol/enhancer solution (Millipore) and exposed using the BioSpectrum 600 system (UVP, Upland, CA, USA).

### 2.8. RNA Isolation and Real-Time PCR for Gene Expression

RNA was extracted from 3T3-L1 cells using TRIzol reagent (Life Technologies, Carlsbad, CA, USA), and cDNA was synthesized using cDNA synthesis kits (Life Technologies).

Real-time PCR cDNA gene expression was detected using the SYBR Green Master kit and a spectrofluorometric thermal cycler (iCycler; Bio-Rad Laboratories, Hercules, CA, USA). Specific primers were designed as shown in Table 1.

### 2.9. Statistical Analysis

Statistical analyses used were one-way ANOVA and Dunnett's post hoc test, and the results were expressed as mean ± standard deviation. *P* values less than 0.05 were considered to be statistically significant.

## 3. Results

### 3.1. Cell Viability and Cytotoxicity of Ginkgolide C in 3T3-L1 Cells

**Figure 1:**
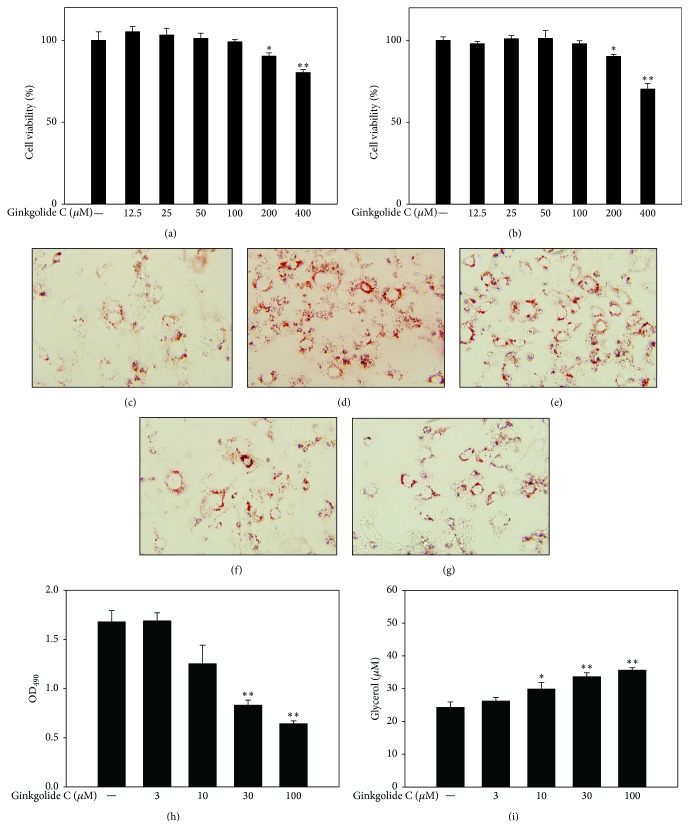
Ginkgolide C suppresses adipogenesis in 3T3-L1 cells. (a) 3T3-L1 preadipocytes and (b) differentiated adipocytes cell viability were evaluated with the MTT assay. Oil red O stain shows (c) differentiated 3T3-L1 cells, (d) treated with ginkgolide C 3 *μ*M, (e) 10 *μ*M, (f) 30 *μ*M, and (g) 100 *μ*M. (h) Cells treated with isopropanol and the lipid accumulation measured at OD 490 nm and (i) glycerol concentrations assayed in supernatants. Data are presented as mean ± SD, *n* = 6. ^*∗*^ *P* < 0.05, ^*∗∗*^ *P* < 0.01, compared with the control group.

The MTT method was used to determine the cytotoxicity of ginkgolide C in 3T3-L1 preadipocyte and differentiated adipocytes. Following treatment for 24 h, ginkgolide C had no significant effect on 3T3-L1 cell viability at concentrations ≤100 *μ*M (Figures 1(a) and 1(b)). Therefore, 3 *μ*M–100 *μ*M ginkgolide C was used in all experiments.

### 3.2. The Effect of Ginkgolide C on Lipid Accumulation in 3T3-L1 Adipocytes

To evaluate the effect of ginkgolide C on adipogenesis, we treated differentiated 3T3-L1 cells with various concentrations of ginkgolide C for 24 h and measured lipid accumulation by oil red O staining. Microscopy evaluation showed that ginkgolide C could significantly decrease oil red O distribution in differentiated 3T3-L1 cells (Figures 1(c)–1(g)). We used isopropanol to release and measure lipid accumulation and found that 10–100 *μ*M but not 3 *μ*M ginkgolide C significantly suppressed lipid accumulation compared with the control group (OD_490_, GC3: 1.68 ± 0.17, *P* = 0.22; GC10: 1.25 ± 0.37, *P* < 0.05; GC30: 0.83 ± 0.11, *P* < 0.01; GC100: 0.64 ± 0.06, *P* < 0.01* versus* control: 1.67 ± 0.23, resp.) (Figure 1(h)). Analysis of the supernatant showed that ginkgolide C also significantly promoted glycerol release (GC3: 26.23 ± 2.15 *μ*M, *P* = 0.42; GC10: 29.88 ± 4.08 *μ*M, *P* < 0.05; GC30: 33.65 ± 2.48 *μ*M, *P* < 0.01; GC100: 35.61 ± 1.60 *μ*M, *P* < 0.01 versus control: 24.26 ± 3.36 *μ*M, resp.) (Figure 1(i)).

### 3.3. The Effect of Ginkgolide C on Transcription Factors of Adipogenesis

**Figure 2:**
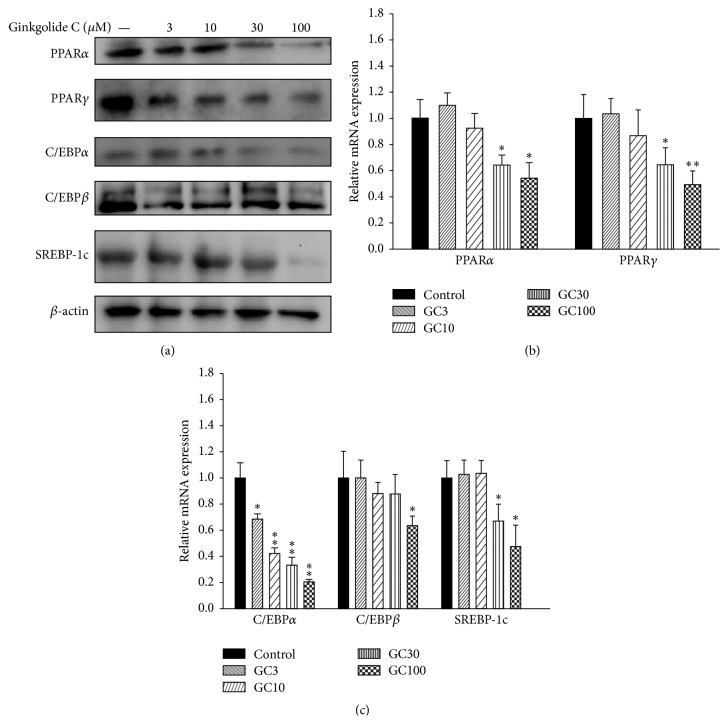
Effects of ginkgolide C (GC) on transcription factors of adipogenesis in differentiated 3T3-L1 adipocytes. (a) Differentiated 3T3-L1 cells were treated with various concentrations of ginkgolide C for 24 h and PPAR-*α*, PPAR-*γ*, C/EBP*α*, C/EBP*β*, and SREBP-1c proteins were detected by western blot (*n* = 3 per group). *β*-actin expression was used as an internal control. ((b) and (c)) Real-time PCR results showing transcription factor gene expression. The fold expression levels were measured relative to the expression of *β*-actin (internal control). Data are presented as the mean ± SD; ^*∗*^ *P* < 0.05, ^*∗∗*^ *P* < 0.01, compared to differentiated 3T3-L1 cells (control group).

Adipocytes can express transcription factors of adipogenesis, including PPAR, C/EBP, and SREBP-1c. Using western blot, we found that differentiated adipocytes treated with ginkgolide C showed suppressed PPAR-*α* and PPAR-*γ* expression compared with the control group (Figure 2(a)). Ginkgolide C also decreased C/EBP*α*, C/EBP*β*, and SREBP-1c expression. We performed real-time PCR assays to detect the mRNA expression of transcription factors of adipogenesis and found that those genes also showed decreased expression compared with the control group (Figures 2(b) and 2(c)).

### 3.4. The Effect of Ginkgolide C on Adipogenesis-Related mRNA and Protein Expression in 3T3-L1 Cells

**Figure 3:**
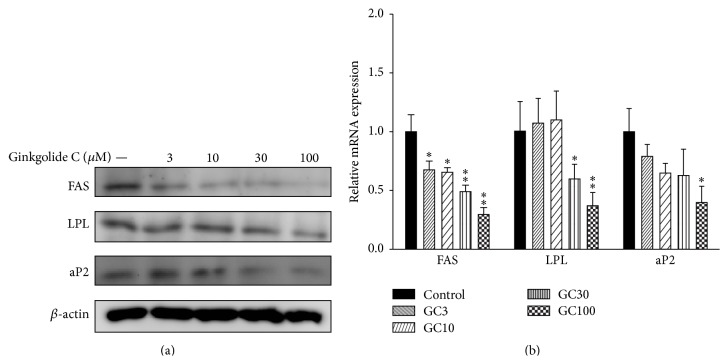
Effects of ginkgolide C (GC) on adipogenesis-related protein expression in differentiated 3T3-L1 adipocytes. (a) The differentiated 3T3-L1 cells were treated with various concentrations of ginkgolide C for 24 h, and FAS, LPL, and aP2 proteins were detected by western blot (*n* = 3 per group). *β*-actin expression was used as an internal control. (b) Real-time PCR results show FAS, LPL, and aP2 gene expression. The fold expression levels were measured relative to the expression of *β*-actin (internal control). Data are presented as the mean ± SD; ^*∗*^ *P* < 0.05, ^*∗∗*^ *P* < 0.01, compared to differentiated 3T3-L1 cells (control group).

To examine the effects of ginkgolide C on adipogenesis, differentiated 3T3-L1 cells were treated with ginkgolide C for 24 h. We found that ginkgolide C could suppress FAS, LPL, and aP2 protein and mRNA expression in a dose-dependent manner (Figures 3(a) and 3(b)).

### 3.5. The Effect of Ginkgolide C on Lipolysis-Related mRNA and Protein Expression in 3T3-L1 Cells

**Figure 4:**
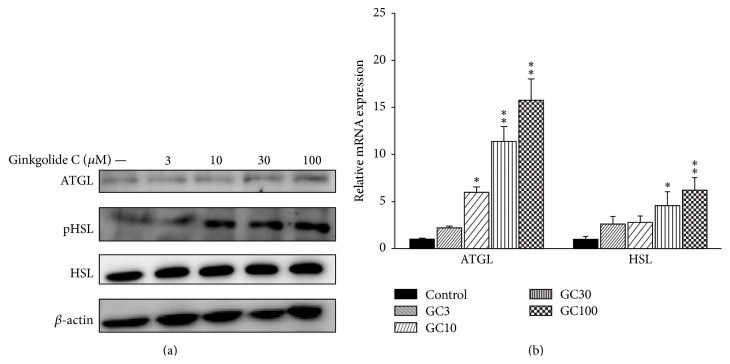
Effects of ginkgolide C (GC) on lipolysis protein expression in differentiated 3T3-L1 adipocytes. (a) Differentiated 3T3-L1 cells were treated with various concentrations of ginkgolide C for 24 h, and ATGL and phosphorylated HSL (pHSL) proteins were detected by western blot (*n* = 3 per group). *β*-actin expression was used as an internal control. (b) Real-time PCR results showing ATGL and HSL gene expression. The fold expression levels were measured relative to the expression of *β*-actin (internal control). Data are presented as the mean ± SD; ^*∗*^ *P* < 0.05, ^*∗∗*^ *P* < 0.01, compared to differentiated 3T3-L1 cells (control group).

Western blotting was used to investigate the effects of ginkgolide C on lipolysis-related protein expression. Ginkgolide C significantly promoted pHSL and expression of ATGL (Figure 4(a)). We also found that ATGL and HSL mRNA expression were significantly increased compared to the control group (Figure 4(b)).

### 3.6. The Effect of Ginkgolide C on the Sirt1 and AMPK Pathway

**Figure 5:**
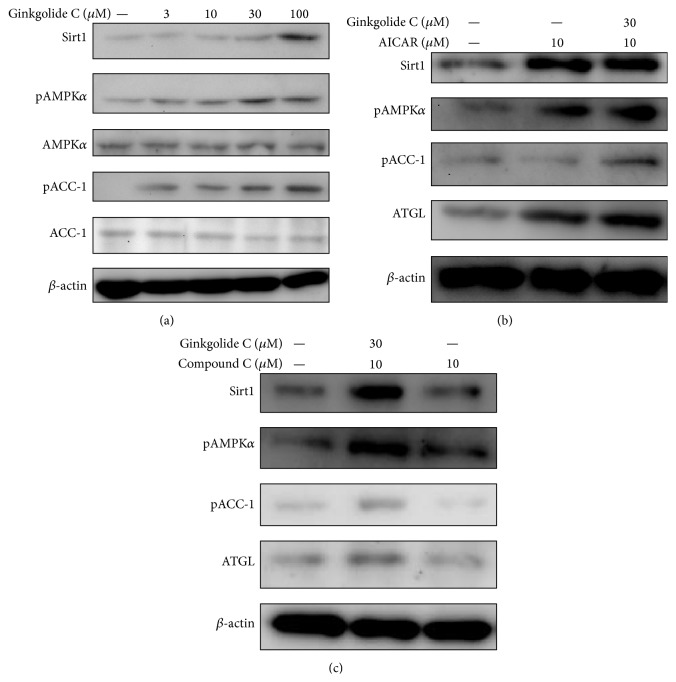
Effects of ginkgolide C on the AMPK pathway in differentiated 3T3-L1 adipocytes. (a) Differentiated 3T3-L1 cells were treated with various concentrations of ginkgolide C for 24 h, and Sirt1, phosphorylation of AMPK*α*, AMPK*α*, phosphorylation of ACC-1, and ACC-1 were detected by western blot (*n* = 3 per group). AMPK*α* or ACC-1 expression was used as an internal control. (b) Ginkgolide C coculture with an AMPK activator (AICAR) affected AMPK phosphorylation and Sirt1 and ATGL protein levels, and (c) ginkgolide C coculture with an AMPK inhibitor (compound C) led to recovered AMPK phosphorylation and Sirt1 and ATGL protein production.

We also investigated whether ginkgolide C could modulate the Sirt1 and AMPK pathway in 3T3-L1 cells. Ginkgolide C significantly promoted Sirt1 production and increased phosphorylation of AMPK*α* and ACC-1 in a concentration-dependent manner (Figure 5(a)). 3T3-L1 treatment with 5-aminoimidazole-4-carboxamide-1-*β*-D-ribofuranoside (AICAR, an AMPK activator) increased Sirt1 levels, phosphorylation of AMPK*α*, and ACC-1 production. Furthermore, coculture of cells with ginkgolide C and AICAR enhanced Sirt1, AMPK activation, and ATGL expression (Figure 5(b)). Of interest, ginkgolide C could recover AMPK activation and Sirt1 and ATGL production when 3T3-L1 cells were cotreated with compound C, an AMPK inhibitor (Figure 5(c)).

## 4. Discussion

In traditional Chinese medicine,* G. biloba* is widely used to treat cardiovascular disease, and* G. biloba* extract (Egb761) is also reported to have neuroprotective effects [19]. Several compounds have been isolated, and ginkgolides A and B have multiple pharmacological activities, including improved platelet aggregation and neuroprotection against cerebral ischemia [13, 20]. In this study, we investigated the antiobesity effect of ginkgolide C in 3T3-L1 adipocytes. Differentiated 3T3-L1 adipocytes that were treated with ginkgolide C showed a reduced accumulation of droplets, a decrease in adipogenesis-related transcription factors, and downregulated expression of fatty acid synthesis enzymes. We also found that ginkgolide C promoted production of lipolysis-related enzymes and regulated AMPK pathway activity.

3T3-L1 preadipocytes differentiate into mature adipocytes that can induce mRNA and protein production of transcription factors associated with fatty acid synthesis [21]. PPARs are important in regulating the metabolism of lipids, carbohydrates, and proteins and involved in cellular differentiation and tumorigenesis [22]. Of the three types of PPARs identified, PPAR*α*, PPAR*β*, and PPAR*γ* [23], PPAR*α* and PPAR*β* are important early transcription factors during adipocyte differentiation [22]. Previous results have shown that ginkgolide C suppresses PPAR*α* gene and protein expression and decreases FAS production [24]. Another work has confirmed that PPAR*α* and PPAR*β* can regulate FAS gene expression [18], but we did not use a PPAR antagonist or agonist to investigate the interaction of PPARs with FAS in ginkgolide C treated adipocytes. Wu et al. found that the pseudoginsenoside F11 attenuates the effect of a PPAR agonist in promoting adipogenesis-related enzyme expression [25], and we plan to examine the relationship between PPARs and ginkgolide C in differentiation.

C/EBP is also closely related to transcription factors associated with the adipocyte differentiation process [21]. Several researchers have found that C/EBP*β* and C/EBP*γ* have rapid effects on the early differentiation of adipocytes and influence PPAR activation [26]. We used mature adipocytes and still detected gene and protein expression of C/EBP*β*. Of interest, ginkgolide C can significantly reduce C/EBP*β* effects, and our results suggested that ginkgolide C can boost fatty acid synthesis in adipocytes.

SREBP-1c is a transcription factor for lipid and cholesterol synthesis [27]. SREBP-1c may bind to the FAS promoter to regulate FAS expression and increase fatty acid synthesis in adipocytes, and SREBP-1c can regulate the activation of cholesterol biosynthesis in hepatocytes [28]. Ginkgolide C significantly suppressed SREBP-1c, PPAR-*γ*, and C/EBP*α* production to block fatty acid synthesis in adipocytes. PPAR-*γ* and C/EBP*α* bind the FAS promoter in adipogenesis and increase LPS and aP2 expression [23]. aP2 is a small lipid-binding protein that is highly expressed in adipose tissue to regulate glucose and lipid metabolism [29] and can bind and transport fatty acids to intracellular targets in mature adipocytes. LPL is released by adipocytes and hydrolyzes lipoprotein particles from very low-density lipoproteins and chylomicrons in the bloodstream, so that the serum contains more free triglycerides [30, 31]. We found that ginkgolide C reduced aP2 production in adipocytes, with the effect of decreasing the transport and metabolism of fatty acids. Many studies have shown that* Ginkgo* improves cardiovascular disease [15]. The current results indicated that ginkgolide C reduces LPL performance and thus serum free fatty acids. We plan to investigate ginkgolide C further to evaluate whether it also improves low-density lipoprotein levels and cholesterol transport in obese mice.

An antiobesity effect will not only include reduced fatty acid synthesis but also involve increased decomposition of triglycerides for reduced lipid accumulation in adipocytes [32]. In the present study, ginkgolide C treatment of mature adipocytes, based on results of staining with oil red O, significantly ameliorated droplet accumulation. We thought that the droplet reduction in adipocytes might be the result of lipid metabolism by lipolysis. ATGL can hydrolyze triacylglycerol to diacylglycerol and one molecule of free fatty acid [33]. Additionally, phosphorylation of HSL allows it to hydrolyze diacylglycerol into monoacylglycerol and a fatty acid [32]. Ginkgolide C could significantly enhance ATGL and phosphorylation of HSL, thus accelerating triglyceride breakdown into glycerol and free fatty acids, resulting in a reduced number and size of lipid droplets in adipocytes, along with increased levels of glycerol released into the culture medium.

Sirt1 plays an important role in modulating the cell cycle for antiaging [34] and also affects the PGC1-*α*/ERR-*α* complex of regulatory transcription factors in adipogenesis [32]. AMPK is a metabolic energy sensor that can sense nutritional stress for regulating glucose and lipid metabolism [13]. Resveratrol can promote Sirt1 expression by activating the AMPK pathway in 3T3-L1 cells and has the potential to improve insulin resistance in diabetes [35]. In obese mice, increased energy intake leads to increased triglyceride storage in adipose tissue and significantly reduced AMPK activity [36]. AMPK phosphorylation induces phosphorylation of its substrate, ACC, which strictly regulates enzymes during fatty acid synthesis for malonyl-CoA production [37]. Of interest, phosphorylated ACC lacks the ability to synthesize fatty acids [38]. In the liver of obese mice, AMPK activation is suppressed, leading to enhanced synthesis of cholesterol and fatty acids [37]. Also of note, Sirt1 activity is suppressed in liver and adipose tissue of obese mice [39]. Thus, upregulated Sirt1 and AMPK are expected to improve lipid metabolism and decrease lipid acceleration. Our experimental results indicated that ginkgolide C can promote Sirt1 expression and phosphorylation of AMPK and also increase phosphorylation of ACC-1 for reducing fatty acid synthesis. Confirming the relationship of AMPK and Sirt1, ginkgolide C could, respectively, improve or recover Sirt1 production and ACC-1 phosphorylation in adipocytes treated with AICAR (AMPK activator) or compound C (AMPK inhibitor). Previous studies found that AMPK activator could increase ATGL expression to accelerate lipolysis in adipocytes [40, 41]. Hashimoto et al. also found that exercise could promote the effect of lipolysis and antiobesity via activated AMPK pathways [42]. The current results suggest that ginkgolide C also can recover ATGL production in 3T3-L1 cells treated with compound C. Hence, ginkgolide C might promote lipolysis by AMPK activation for increased ATGL production to hydrolyze triglycerides in adipocytes.

## 5. Conclusion

Overweight and obesity are not only the result of adipocyte proliferation but also the result of excess lipid accumulation in liver and adipocyte tissue. Obesity leads to metabolic syndrome and insulin resistance and causes type II diabetes, hypertension, and high cholesterol. We confirmed that ginkgolide C significantly inhibited transcription factors and enzymes associated with adipogenesis and promoted Sirt1/AMPK activity to increase lipolysis in differentiated adipocytes. We suggest that ginkgolide C holds potential for improving metabolic syndrome and insulin resistance.

## Figures and Tables

**Table 1:** Primers used in the experiments.

Gene	Primers	(5′-3′ sequence)	GenBank accession number	Product size (bp)
PPAR-*α*	Forward	GGAGCGTTGTCTGGAGGTT	NM_005036	116
	Reverse	GAAGTGGTGGCTAAGTTGTTGA		

PPAR-*γ*	Forward	GATGACAGCGACTTGGCAAT	NM_138712	107
	Reverse	TGTAGCAGGTTGTCTTGAATGT		

C/EBP*α*	Forward	GACTTGGTGCGTCTAAGATGAG	NM_001287424	149
	Reverse	TAGGCATTGGAGCGGTGAG		

C/EBP*β*	Forward	GTCCAAACCAACCGCACAT	NM_005194	106
	Reverse	CAGAGGGAGAAGCAGAGAGTT		

SREBP-1c	Forward	CTGTTGGTGCTCGTCTCCT	NM_004176	98
	Reverse	TTGCGATGCCTCCAGAAGTA		

FAS	Forward	ATCCTGGCTGACGAAGACTC	AY451392.1	148
	Reverse	TGCTGCTGAGGTTGGAGAG		

LPL	Forward	GGCTCTGCTTGAGTTGTAGAA	NM_008509.2	121
	Reverse	GGCATCTGAGAACGAGTCTTC		

aP2	Forward	AATGAGCAAGTGGCAAGA	NM_011547.4	147
	Reverse	GGTCAAGCAACTCTGGAT		

ATGL	Reverse	AGACAACCTGCCACTCTATGA	AY894804.1	131
	Forward	ACTGGATGCTGGTGTTGGT		

HSL	Reverse	GCTCACGGTCACCATCTCA	NM_005357	103
	Forward	CTCCTCACTGTCCTGTCCTTC		

*β*-actin	Forward	AAGACCTCTATGCCAACACAGT	NM_007393.3	92
	Reverse	AGCCAGAGCAGTAATCTCCTTC		
