# Corrigendum to “Ginkgolide C Suppresses Adipogenesis in 3T3-L1 Adipocytes via the AMPK Signaling Pathway”

**DOI:** 10.1155/2017/4293429

**Published:** 2017-12-03

**Authors:** Chian-Jiun Liou, Xuan-Yu Lai, Ya-Ling Chen, Chia-Ling Wang, Ciao-Han Wei, Wen-Chung Huang

**Affiliations:** ^1^Department of Nursing, Chang Gung University of Science and Technology, 261 Wen-Hwa 1st Road, Kwei-Shan, Taoyuan, Taiwan; ^2^Department of Nutrition and Health Sciences, Chang Gung University of Science and Technology, 261 Wen-Hwa 1st Road, Kwei-Shan, Taoyuan, Taiwan; ^3^Graduate Institute of Health Industry Technology, Chang Gung University of Science and Technology, Kwei-Shan, Taoyuan, Taiwan; ^4^Research Center for Industry of Human Ecology, Chang Gung University of Science and Technology, Kwei-Shan, Taoyuan, Taiwan

In the article titled “Ginkgolide C Suppresses Adipogenesis in 3T3-L1 Adipocytes via the AMPK Signaling Pathway” [1] the statement “Ginkgolide C, isolated from Ginkgo biloba leaves, is a flavone reported to have multiple biological functions, from decreased platelet aggregation to ameliorating Alzheimer disease” in the Abstract section should be changed to “Ginkgolide C, isolated from Ginkgo biloba leaves, is a diterpene lactone derivative reported to have multiple biological functions, from decreased platelet aggregation to ameliorating Alzheimer disease.”
